# Term-BLAST-like alignment tool for concept recognition in noisy clinical texts

**DOI:** 10.1093/bioinformatics/btad716

**Published:** 2023-11-24

**Authors:** Tudor Groza, Honghan Wu, Marcel E Dinger, Daniel Danis, Coleman Hilton, Anita Bagley, Jon R Davids, Ling Luo, Zhiyong Lu, Peter N Robinson

**Affiliations:** Rare Care Centre, Perth Children’s Hospital, Nedlands, WA 6009, Australia; Genetics and Rare Diseases Program, Telethon Kids Institute, Nedlands, WA 6009, Australia; Institute of Health Informatics, University College London, London WC1E 6BT, United Kingdom; Pryzm Health, Sydney, NSW 2089, Australia; School of Life and Environmental Sciences, Faculty of Science, University of Sydney, NSW 2006, Australia; The Jackson Laboratory for Genomic Medicine, Farmington, CT 06032, United States; Shriners Children’s Corporate Headquarters, Tampa, FL 33607, United States; Shriners Children's Northern California, Sacramento, CA 95817, United States; Shriners Children's Northern California, Sacramento, CA 95817, United States; National Center for Biotechnology Information, National Library of Medicine, National Institutes of Health, Bethesda, MD 20894, United States; National Center for Biotechnology Information, National Library of Medicine, National Institutes of Health, Bethesda, MD 20894, United States; The Jackson Laboratory for Genomic Medicine, Farmington, CT 06032, United States; Institute for Systems Genomics, University of Connecticut, Farmington, CT 06032, United States

## Abstract

**Motivation:**

Methods for concept recognition (CR) in clinical texts have largely been tested on abstracts or articles from the medical literature. However, texts from electronic health records (EHRs) frequently contain spelling errors, abbreviations, and other nonstandard ways of representing clinical concepts.

**Results:**

Here, we present a method inspired by the BLAST algorithm for biosequence alignment that screens texts for potential matches on the basis of matching k-mer counts and scores candidates based on conformance to typical patterns of spelling errors derived from 2.9 million clinical notes. Our method, the Term-BLAST-like alignment tool (TBLAT) leverages a gold standard corpus for typographical errors to implement a sequence alignment-inspired method for efficient entity linkage. We present a comprehensive experimental comparison of TBLAT with five widely used tools. Experimental results show an increase of 10% in recall on scientific publications and 20% increase in recall on EHR records (when compared against the next best method), hence supporting a significant enhancement of the entity linking task. The method can be used stand-alone or as a complement to existing approaches.

**Availability and implementation:**

Fenominal is a Java library that implements TBLAT for named CR of Human Phenotype Ontology terms and is available at https://github.com/monarch-initiative/fenominal under the GNU General Public License v3.0.

## 1 Introduction

Deep phenotyping of patients suspected of or diagnosed with a rare disease (RD) has become standard practice in the last ten years. In this context, deep phenotyping refers to the precise and comprehensive annotation of phenotypic abnormalities in a computer-readable format. Computational deep phenotyping relies on community-curated and -maintained ontologies that support the description of patient phenotype profiles via ontological concepts. The clinical utility of deep phenotyping has been showcased repeatedly over the years, in particular, in data sharing (Taruscio *et al.* 2015, Boycott *et al.* 2022, Jacobsen *et al.* 2022) and clinical variant prioritization and interpretation (Smedley *et al.* 2016, Clark *et al.* 2018, Son *et al.* 2018).

The Human Phenotype Ontology (HPO) provides the most comprehensive resource for computational deep phenotyping and has become the de facto standard for encoding phenotypes in the RD domain, for both disease definitions as well as patient profiles to aid genomic diagnostics (Robinson *et al.* 2008, Köhler *et al.* 2009, Smedley *et al.* 2021). The ontology, maintained by the Monarch Initiative (Shefchek *et al.* 2020), provides a set of more than 16 500 terms describing human phenotypic abnormalities, arranged as a hierarchy with the most specific terms furthest from the root term, “Phenotypic abnormality.”

The process of deep phenotyping can be slow, tedious, and error prone if performed manually, which can be a substantial barrier to uptake from clinicians. In recent years, however, natural language processing tools have been developed and have replaced or supplemented manual encoding of phenotype profiles (Arbabi *et al.* 2019, Deisseroth *et al.* 2019). These tools implement various approaches to address the challenges governing the phenotype concept recognition (CR) task such as ambiguity, use of metaphorical expressions, negation, and complex or nested sentence structures (Groza *et al.* 2015).

There are two groups of approaches to phenotype CR: “traditional” dictionary-based and machine learning (ML)-based methods (Gorinski *et al.* 2019). Dictionary-based approaches rely on creating and using inverted indexes from the tokens composing HPO concepts (usually both labels and synonyms), are relatively fast, and provide packaging options that enable deployment in resource-constrained environments, such as typical clinical practices. They achieve high precision at the expense of lower recall rates and tend to struggle with identifying concepts that consist of unseen tokens. The most representative tools in this category are: NCBO Annotator (Jonquet *et al.* 2009), ZOOMA (Kapushesky *et al.* 2012), OBO Annotator (Taboada *et al.* 2014), SORTA (Pang *et al.* 2015), Doc2HPO (Liu *et al.* 2019), ClinPhen (Deisseroth *et al.* 2019), and the Monarch Initiative annotator (Shefchek *et al.* 2020). ML-based approaches are less common in phenotype CR than in other domains where multiple well-established gold standard corpora exist. Two recent attempts, however, have showcased the use of the latest developments in Deep Learning algorithms and models with application to phenotype CR and have achieved state-of-the-art results. Arbabi *et al.* (2019) have developed a neural concept recognizer using a convolutional neural network-based neural dictionary model and tested it successfully on both scientific abstracts and medical notes. PhenoTagger (Luo *et al.* 2021), which is also the current best of breed approach, was developed as a hybrid method that combines dictionary tagging with a BioBERT-based tagger (Lee *et al.* 2019) to efficiently identify HPO concepts—including unseen synonyms and nested subconcepts. While they provide superior experimental results, ML methods impose a heavy computational footprint, which makes them less feasible for deployment outside of server or cloud environments.

This article focuses on a challenge largely ignored or assumed to be implicitly solved by the existing phenotype CR tools—i.e. typographical errors often encountered in real-world clinical settings. Typographical errors can be seen as a subclass of lexical variation in the context of the CR task. They are significantly less structured than standard lexical variation, with the semantics of the tokens being harder to predict and interpret. For example, the tokens *short* and *shortening* in *Short phalanx of finger* and *Shortening of the finger phalanges*, respectively (HP:0009803) have a well-defined linguistic interpretation (e.g. in terms of the root/lemma and part of speech tag) and can both be folded into the token *short*. The same can be stated for *shorter* and *shortest*. Tokens such as *shoret* or *shprt*, on the other hand, are more difficult to interpret automatically, although a human would instantly recognize them as common typographical errors of the token *short*. In this article, we investigate the accuracy of current phenotype CR methods in the presence of typographical errors.

The contributions of this article are 3-fold. Firstly, we make available a gold standard corpus for typographical errors with relevance to clinical phenotyping. Secondly, we propose a novel method to perform fast and accurate token-level matching with applications to entity linkage in the presence of typographical errors. The method is conceptually inspired from sequence alignment algorithms—Basic Local Alignment Search Tool (BLAST) (Altschul *et al.* 1990) and FASTA (Lipman and Pearson 1985)—and uses an underlying scoring matrix, which was built from 3-mers belonging to tokens present in the gold standard. Conceptually, our approach is similar to the one proposed by Krauthammer *et al.* (2000), while methodologically it is completely different. Thirdly, we perform a systematic evaluation of our approach and the state-of-the-art phenotype CR tools. Our study represents an extension to the work of Kim *et al.* (2022) with a focus on assessing the versatility of existing phenotype CR methods in the presence of typographical errors. The results show that our method enhances significantly the entity linkage task. Moreover, both the scoring matrix and the methodology can be used to augment existing dictionary or ML-based tools.

## 2 Materials and methods

We present an algorithm for CR of HPO terms in the presence of spelling errors, alternate spellings, partial matches, and altered orderings of the tokens that make up the term.

### 2.1 Term-BLAST-like alignment tool

**Figure 1.**
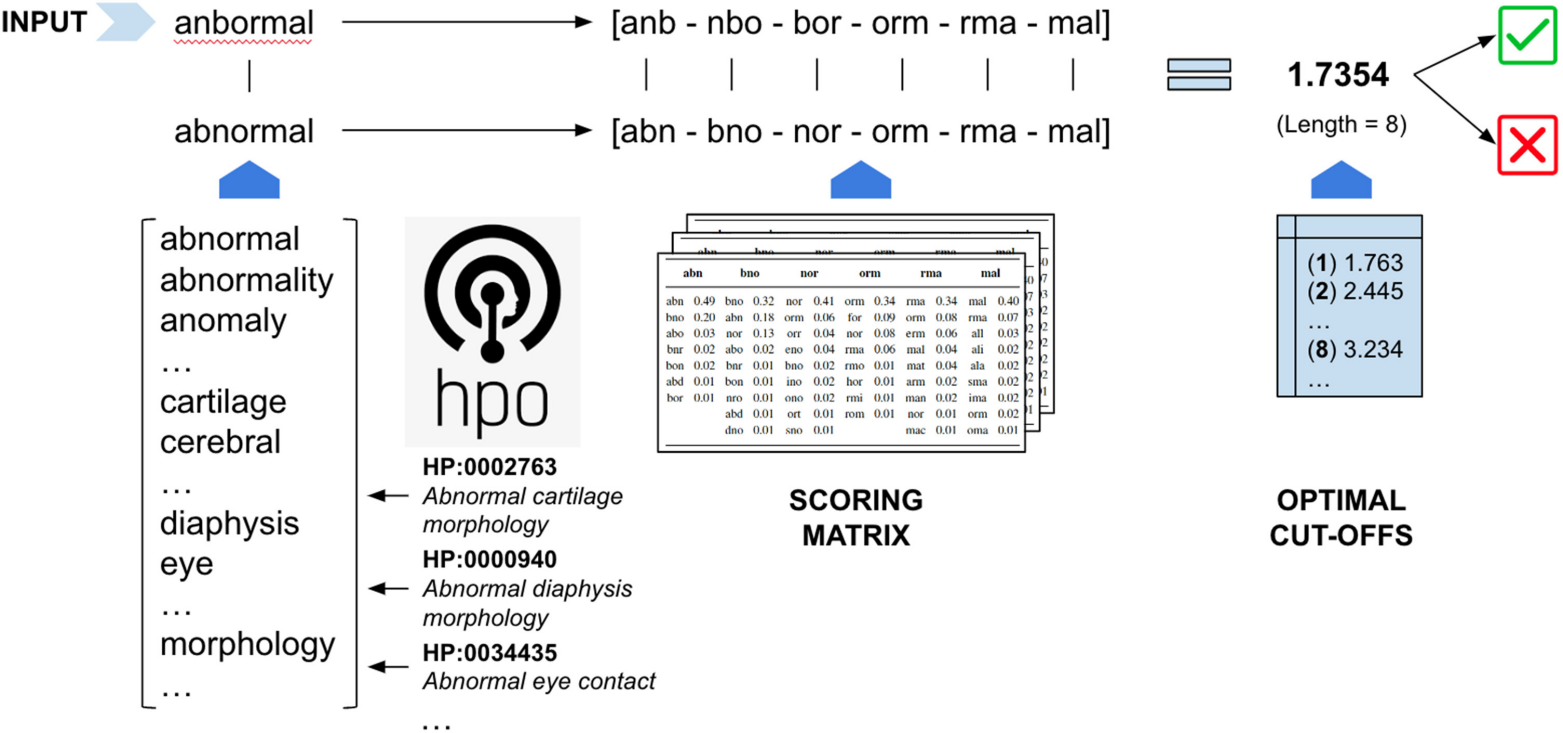
High-level overview of the proposed term-BLAST-like alignment approach.

The BLAST finds regions of local similarity between sequences without performing a comprehensive search of every residue against each other. Instead, BLAST uses short word segments to create alignment seeds that are extended if a specified match threshold is exceeded (Altschul *et al.* 1990). The BLASTP algorithm is an extension of BLAST for protein sequences that uses amino-acid scoring matrices that capture evolutionary information to estimate the probability of any given amino-acid substitution (Altschul *et al.* 1997). Here, we present the Term-BLAST-like alignment tool (TBLAT). TBLAT adapts the general strategy of the BLAST algorithm to CR in potentially noisy clinical texts. A high-level overview of the approach is depicted in Fig. 1. The algorithm compares a given candidate against the list of tokens compiled from a given ontology—in our case, the HPO—by computing an alignment score between the sets of 3-mers composing the tokens. A scoring matrix underpins the computation of the score and a final decision is proposed using precomputed optimal cut-offs and the length of the original candidate token. Each of these steps is detailed in the following sections.

### 2.2 Human phenotype ontology

In this work, we apply the TBLAT method for recognition of HPO concepts (terms). The HPO version 02–2022 (February 2022) was used, which contains 16 801 terms and 18 999 exact synonyms. For instance, the term “Cataract” (HP:0000518) has five synonyms including “Lens opacity.”

### 2.3 Data

The starting point of our investigation was the creation of a gold standard corpus of clinical typographical errors derived from a real-world setting. The foundation of our corpus is represented by a set of 2 692 451 clinical notes collected from multiple individual clinics in a primary care setting in Australia and authored by 22 doctors. Given the heterogeneity of both the environment, as well as of the individual primary care physician practices, the notes did not follow a structured and uniform authoring template. We extracted the notes from the clinics using a data extraction tool available within the general practice medical records software. The same tool, enabled an initial deidentification, by removing personal health identification elements, such as names, addresses, phone numbers, dates of birth, and other government identifiers.

The deidentified notes were processed using a standard spaCy text processing pipeline (sentence splitter and tokenizer), available at https://spacy.io/. This resulted in sentences and a total of 159 236 294 tokens forming them (700 475 of which were unique)—see Table 1. All unique tokens were cleansed of punctuation marks (e.g. “,”, “;”, “:”, “>”, “<”) and those containing special characters (e.g. “@,” “+”). Tokens shorter than four characters were removed.

**Table 1.** Clinical token corpus statistics.

Number of clinical notes	2 692 451
Number of tokens	159 236 294
Unique tokens	700 475
**(GS)** Unique “canonical” tokens (length > 4)	256 005
**(GS)** Unique typographical errors	51 337
Unique tokens in ontology concepts	6862
Ontology tokens in gold standard	4769
Ontology tokens with typographical errors	3858

We assumed that a substantial proportion of the token set represented spelling errors or other nonstandard spellings. Therefore, we used the GoogleNews 3 million words and phrases word2vec model (Mikolov *et al.* 2013) to find matches for each of the 700 475 unique tokens. The consolidation was performed by picking the top most similar token and regrouping them around tokens with the highest number of occurrences. This resulted in 256 005 unique “canonical” tokens and 51 337 typographical errors, where a “canonical” token is assumed to be the “correct” word. Note that this canonical token does not refer to a root or lemma of a set of words, but rather to the correct spelling of a set of misspelled tokens. An example of a consolidated row of tokens is: actions::[actions, tactions, actons, acions, …] (where actions is the “canonical” token). All rows consisting of sets of nonidentical tokens were validated by one of the authors (T.G.), who applied a best-judgment call of whether a pair of tokens could represent a realistic typographical error or not. This “gold standard” is used as a basis for all further experiments and is available for download at: https://github.com/monarch-initiative/fenominal.

To gain a better understanding of the nature of the typographical errors, we split the tokens into their component 3-mers, recorded the transition from one 3-mer in the “canonical” token to one 3-mer in the typographical error (at the same index) and classified it into one of the six groups described and exemplified below. A 3-mer represents the decomposition of a word into all orderly occurring substrings of length 3, in our case with overlap—e.g. actions::[act, cti, tio, ion, ons]. Also, note: the “_” character below denotes “any other character.”

- “Shift” errors (42.25%) represent a shift to the left or to the right of two out of the three characters in the 3-mer, e.g. met→ame or met→eta. Example: abnormal→abbnormal.
- “Single replacement” errors (18.9%) denote a replacement of one of the three characters in the 3-mer with another, e.g. tar→t_r or tar→_ar or tar→ta_. Example: abnormal→annormal.
- “Double replacement” errors (8.79%) are the same as “single replacement,” however, covering two characters, e.g. tar→t__ or tar→__r or tar→_a_. Example: abnormal→andnormal.
- “Gap shift” (5.63%) model an insertion of a random character in the middle of the 3-mer, e.g. tar→t_a or tar→a_r. Example: abnormal→abdnormal.
- “Inversions” (3.02%) are a form of “gap shifts” where two characters in the 3-mer are swapped around, e.g. tar→atr or tar→tra. Example: abnormal→anbormal.

All other errors, denoting random 3-mer transitions, were placed in an “Other” category covering 21.41% of the total number of errors (e.g. bno→atr). For example, abnormal→itormal.

Finally, given our focus on HPO phenotypes, we summarize in Table 1 the coverage in the gold standard of the tokens composing labels and synonyms defined by HPO concepts. All HPO labels and synonyms consist of 6862 unique tokens (unigrams), once stop words were removed (the list of all stop words used by TBLAT is available in Supplementary Section S1) and the same cleaning procedure was applied as in the case of clinical notes. Of the total set, 4769 tokens are present as “canonical” tokens in the gold standard (i.e. the corpus of 256 005 canonical tokens) with 3858 being associated in the gold standard with typographical errors. The 2093 tokens not present in the gold standard, and hence in the clinical notes corpus, represent predominantly medical terms with a length larger than 15 (e.g. “glutarylcarnitine”) and highly specialized phenotypes—e.g. “pseudohypoaldosteronism.”

### 2.4 Scoring matrix

Inspired conceptually by the method used to construct the PAM (Point Accepted Mutation) and BLOSUM (BLOcks SUbstitution Matrix) substitution matrices that underpin the most widely used approaches in sequence alignment, we built a similar scoring matrix aimed at encoding the probability of one 3-mer, belonging to a “canonical” (*correct*) token, transitioning into another 3-mer, belonging to a typographical error, given the gold standard corpus described in Section 2.3 as background. Below are the steps applied to each row in the gold standard corpus to build the matrix:

1. Generate 3-mers for the “canonical” token and all associated typographical errors. For example, the token abnormal would be represented by the list [*abn*, *bno*, *nor*, *orm*, *rma*, *mal*]. Similarly, a typographical error abnrmal would be represented by [*abn*, *bnr*, *nrm*, *rma*, *mal*].
2. Aggregate the frequency of position-based transitions across the entire corpus for all 3-mers contained by “canonical” tokens to all other 3-mers contained by typographical errors. For example, given the pair abnormal—abnrmal, the 3-mers belonging to the “canonical” token abnormal would record the following position-based transitions: (i) [0] *abn* →*abn*; (ii) [1] *bno* →*bnr*; (iii) [2] *nor* →*nrm*; (iv) [3] *orm* →*rma*; (v) [4] *rma* →*mal*

There are a few remarks worth noting. Firstly, while the consolidation described above includes the identical token (i.e. actions is associated to actions in the gold standard), the identity association was removed when computing the frequencies (i.e. only typographical errors were included in the frequency calculation). Secondly, the 3-mer transitions were computed from left to right—i.e. from the “canonical” token to one of the typographical errors and always driven by the indices of the 3-mers composing the “canonical” token—i.e. 3-mer at index 0 in the “canonical” token against 3-mer at index 0 in the typographical error, 3-mer at index 1 against 3-mer at index 1, and so on. This leads to three possible scenarios, subject to the difference in length between the “canonical” token and the typographical error: all 3-mers in the “canonical” token have a transition into 3-mers in the typographical error if the two have the same length; some 3-mers in the “canonical” token do not have a transition if “canonical” token is longer than the typographical error and vice-versa; and some 3-mers in the typographical error do not have a transition if “canonical” token is shorter than the typographical error.

For example, the state transitions for the 3-mer abn are: abn: 0.492562; bno: 0.203306; abo: 0.034711; bnr: 0.021488; bon: 0.021488; abd: 0.016529; bor: 0.01157. To complete the final scoring matrix, we removed all transitions with a value of less than 0.01. The scoring matrix is available for download at: https://github.com/monarch-initiative/fenominal.

### 2.5 Token-level alignment score

The alignment score defined by the standard sequence alignment algorithms (BLAST or FASTA) usually sums up the values listed in a substitution matrix when considering the comparison between nucleotides or amino acids in a query sequence and sequences in a database. A second component of the score is a gap penalty aimed to penalize noncontiguous alignments between the two sequences.

Our method uses the same principles, applied however in a different manner. The alignment score sums up values for 3-mer transitions found between the query and the candidate tokens using the scoring matrix. In addition, it also caters for scenarios when such transitions are missing, while penalizing the lack of coverage for all 3-mers in the candidate token. More concretely, the frequency values of the error types (e.g. “shift,” “single replacement”—see Section 2.3) are used as a penalty in conjunction with identity transition values to fill in areas not covered by the scoring matrix because of the lack of data in the gold corpus.

### 2.6 HPO-focused token score distributions

To understand how “close” an alignment is between a query sequence and a sequence retrieved from a background database, algorithms like BLAST recommend interpreting the score by observing its percentile placement in the context of the overall score distribution, which is computed by moving the query sequence in all possible positions against candidate sequences retrieved from the database.

We adopted the concept of computing score distributions to support the interpretation of the score by using gold standard listed in Table 1. Our focus was phenotype concepts defined by HPO, and as such, we considered the tokens contained within all HPO classes as target dictionary—i.e. the 4769 ontology tokens that exist as “canonical” tokens in our dataset, out of the total of 6862 possible unique ontology tokens (see Table 1). Note that in our case, HPO acts as the proxy for the background database used by BLAST. For each token, we aimed to capture the entire space an alignment score could take if all 3-mers composing the token would systematically transition through all possible values in the scoring matrix. The actual score was computed as per the BLAST method, by summing up the values recorded in the substitution matrix between pairs of 3-mers representing a transition from a “canonical” token to a typographical error. The procedure leads to a score distribution, which enables us to assign to a random alignment score the same interpretation used in BLAST.

To exemplify the score computation, Table 2 lists the state transitions for all 3-mers composing the token abnormal. A maximal alignment score is achieved by summing up all identity state transitions, i.e.:

**Table 2.** State transition table for all 3-mers composing the token abnormal.

abn		bno		nor		orm		rma		mal	
abn	0.49	bno	0.32	nor	0.41	orm	0.34	rma	0.34	mal	0.40
bno	0.20	abn	0.18	orm	0.06	for	0.09	orm	0.08	rma	0.07
abo	0.03	nor	0.13	orr	0.04	nor	0.08	erm	0.06	all	0.03
bnr	0.02	abo	0.02	eno	0.04	rma	0.06	mal	0.04	ali	0.02
bon	0.02	bnr	0.01	bno	0.02	rmo	0.01	mat	0.04	ala	0.02
abd	0.01	bon	0.01	ino	0.02	hor	0.01	arm	0.02	sma	0.02
bor	0.01	nro	0.01	ono	0.02	rmi	0.01	man	0.02	ima	0.02
		abd	0.01	ort	0.01	rom	0.01	nor	0.01	orm	0.02
		dno	0.01	sno	0.01			mac	0.01	oma	0.01

- abn + bno + nor + orm + rma + mal = 0.49 + 0.32 + 0.41 + 0.34 + 0.34 +0.40 = 2.3

Similarly, an alignment score computed over a series of random transitions would be:

- abo + bno + nor + orm + rma + mal = 0.03 + 0.32 + 0.41 + 0.34 + 0.34 + 0.40 = 1.84

As such, for the token abnormal, the score distribution ranges from an alignment score of 0.06 (5% alignment score) to 2.3 (100% alignment score).

Supplementary Section S2 in the material provides a comprehensive overview of the coverage across all 4769 “canonical” ontology tokens given the underlying score distributions when the tokens are grouped according to their length. This is relevant in the context of establishing a cut-off, which drives the control of the sensitivity and specificity of the matching strategy. In principle, subject to the underlying application, this could be set dynamically or fixed using some principles. Here, we showcase the impact of the length of the token over the coverage of typographical errors and provide guidance on how it can be used to generalize the alignment score.

There are two conclusions that can be drawn from the shape of the trends in Supplementary Section S2. Firstly, shorter tokens are more volatile and require a lower value for the alignment score to have a reasonable coverage. This is evident for lengths of 5–8, where the coverage drops almost linearly. From a length of 9, the alignment score becomes more stable, while from a length of 10 and upwards it becomes clear that a fixed cut-off set around the 70th percentile is justifiable.

**Figure 2.**
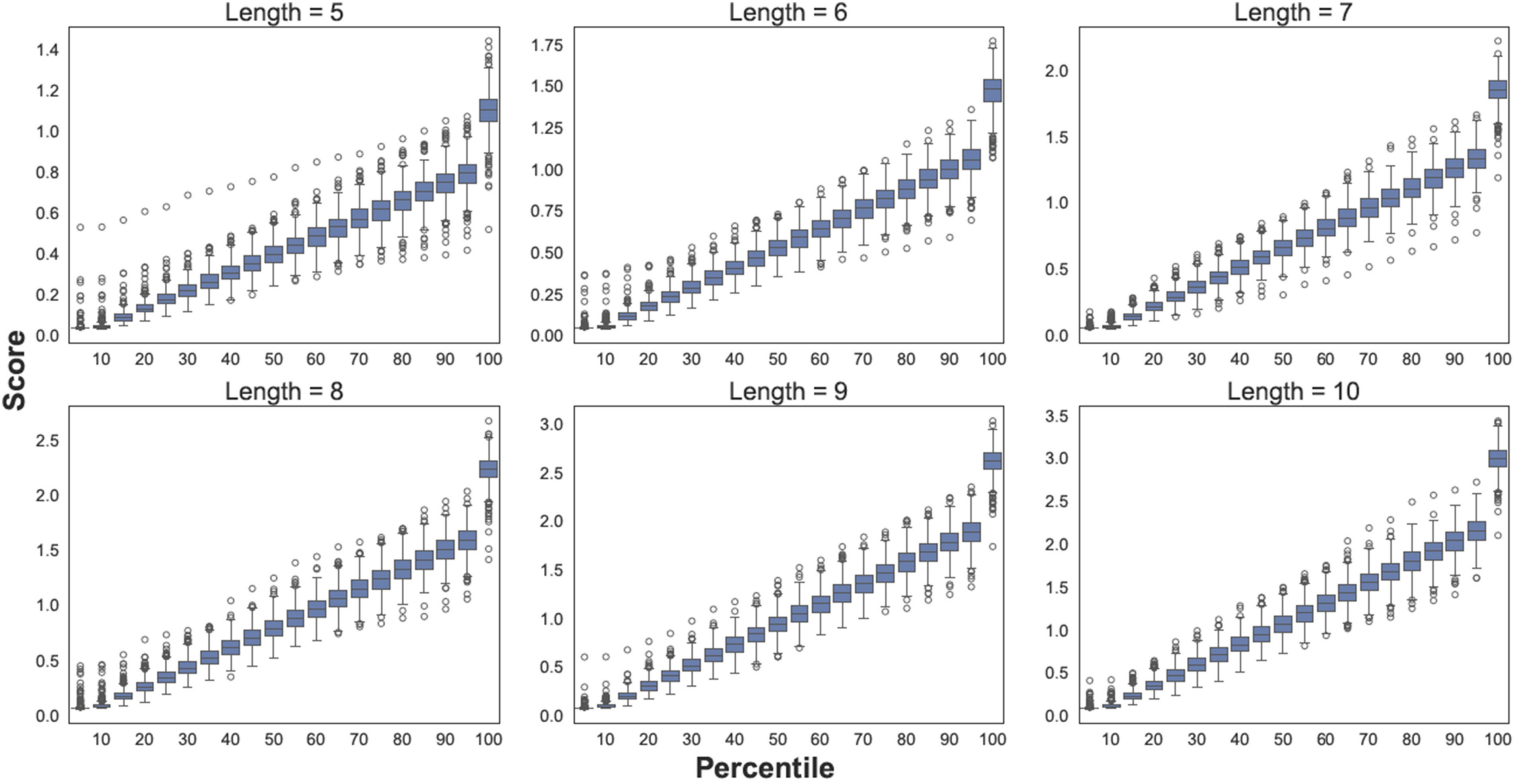
Distribution of alignment scores for all tokens derived from the HPO. The box plots at 5%, 10%, 15%, …, 100% are shown (Note: odd numbers are omitted from the *X*-axis label). Each box plot represents the corresponding percentile scores for the tokens. For instance, the 5% alignment score for “abdomen” is 0.05497, and this score, as well as the corresponding score for other tokens, is used to generate the box plots.

The actual value of the alignment score cut-off can be deduced using the distributions depicted in Fig. 2. The boxplots capture the scores and alignment percentiles for token lengths of 5–10. The recommendation is to choose a target percentile based on the desired coverage—while noting that a larger coverage will have an impact on precision—and then find the associated score by looking up the minimal value of the score distribution for the selected percentile. The evaluation discussed in the next section provides concrete usage scenarios for cut-offs.

### 2.7 Software availability

*fenominal* (Fabulous phENOtype MINing ALgorithm) is a Java 17 software library that implements the TBLAT algorithm. *fenominal* aims to provide a module-based framework to perform HPO CR, with a particular focus on applications in resource-constraint clinical-sensitive environments—i.e. clinical settings where the data can only be accessed inside the organization’s firewall. The package includes a command-line interface that can be used to parse text files. Detailed instructions for use are available. The software is available under the GNU General Public License v3.0 at https://github.com/monarch-initiative/fenominal. *fenominal* is also available on Maven Central at https://search.maven.org/artifact/org.monarchinitiative.fenominal/fenominal (version 0.7.9 was current at the time of this writing).

### 2.8 Experimental setup

#### 2.8.1 Tools

The evaluation was carried out using the following tools:

**Doc2HPO** (Liu *et al.* 2019)—via API, with default parameters, during 10–13 August 2022, as per instructions provided at https://github.com/stormliucong/doc2hpo;

**ClinPheno** (Deisseroth *et al.* 2019)—MacOS download, version available on 10 August 2022 from http://bejerano.stanford.edu/clinphen/;

**Monarch Initiative annotator** (Shefchek *et al.* 2020)—via API, with default parameters (match over five characters long), during 10–13 August 2022;

**NCBO Annotator** (Jonquet *et al.* 2009)—via API, with default parameters, during 10–13 August 2022;

**PhenoTagger** (Luo *et al.* 2021)—release v1.1 downloaded from https://github.com/ncbi-nlp/PhenoTagger with models v1.1 downloaded from https://ftp.ncbi.nlm.nih.gov/pub/lu/PhenoTagger/models_v1.1.zip and installed as per the instructions available on 10 August 2022; runs were executed with default parameters.

#### 2.8.2 Corpora

We used three corpora in our experiments, all in conjunction with HPO version 02–2022 from February 2022.

*GSC+*: This is a manually curated dataset that consists of 228 manually annotated abstracts of scientific publications (Lobo *et al.* 2017). To ensure consistency, we realigned the annotations to HPO version 02–2022 by replacing retired HPO IDs with the most up-to-date IDs specified via the alt_id property. The operation left no orphan annotations in the corpus. GSC+ was used in two settings:

- GSC+ (baseline)—the original corpus, to record a baseline against a perfectly clean set of text entries
- GSC+ (typo)—the original corpus with all tokens present in the set of ontology tokens with typographical errors—i.e. the 3858 mentioned in Table 1 replaced systematically with the associated typographical errors.

Table 3 lists the high-level statistics of GSC+ (baseline). As a note, “canonical” tokens in the table refer to tokens contained by gold standard corpus of typographical errors (i.e. the set of 256 006 tokens from Table 1) that had nonidentical typographical errors associated with them. The most relevant aspect in Table 3 is the percentage of corpus annotations containing tokens that can be used to perform an appropriate validation—i.e. 85.2%. The annotations comprised a total of 3685 “canonical” tokens—i.e. 64.4% of the entire set of tokens—with 571 “canonical” tokens being unique—i.e. 55.2% of the total set of unique tokens. Similarly, Table 4 lists the high-level statistics of GSC+ (typo), generated by systematically replacing the 3685 “canonical” tokens in GSC+ (baseline) with typographical errors. This resulted in 61 902 annotations (after removing those that did not contain a “canonical” tokens) comprising 95 191 typographical error tokens.

**Table 3.** Baseline corpora statistics.

	GSC+	EHR
Number of documents	228	100
Number of annotations	2773	1815
Unique HPO concepts	461	252
Total number of tokens in the corpus	5724	59 470
Unique tokens in the corpus	1035	5672
Annotations containing “canonical” tokens	2362 (85.2%)	1450 (79.9%)
Total “canonical” tokens	3685 (64.4%)	1095 (19.3%)
Unique “canonical” tokens	571 (55.2%)	260 (23.7%)

**Table 4.** Typo corpora statistics.

	GSC+	EHR
Number of documents	228	100
Unique HPO concepts	461	252
Annotations containing typographical errors	61 902	57 789
Total typographical error tokens	95 191	37 242

*Electronic Health Record (EHR)*: A set of 100 anonymized clinical notes from Shriners Children’s—corpus named EHR hereafter. Tables 3 and 4 also list the statistics of the EHR corpus containing 100 clinical notes from Shriner’s Children’s patients with a diagnosis of cerebral palsy. The source notes were retrieved from the EHR in rich text format and decompressed using the system vendor’s APIs on their propriety compression format. Once decompressed, notes were stripped of their embedded rich text formatting commands. The final step taken in data preparation was to use structured data from the health record to search and replace key PHI terms from the clinical notes, including medical record number, names, and addresses, thus yielding a limited dataset suitable for research.

We chose to introduce this second corpus in order to diversify both the type of textual entries, as well as the underlying domain. The corpus was manually annotated by T.G. and P.N.R., which led to 1815 annotations using 252 unique HPO concepts. Although smaller in number of documents, the corpus is significantly larger in total number of tokens (59 470), as well as the unique number of tokens (5672), while maintaining a comparable coverage of “canonical” tokens across the HPO annotations (79.9%). The overlap between GSC+ and the EHR corpora was limited—51 unique HPO concepts and 101 unique “canonical” tokens.

*GenTypo*: Finally, the GenTypo corpus was generated from the remainder of 2093 ontology tokens not present in the gold standard token corpus listed in Table 1. The aim of using this second corpus is to test the versatility of the method on tokens not seen before in establishing the alignment score distributions and cut-offs. GenTypo was created by systematically introducing shift, single replacement, and inversion typographical errors into each of the 2093 ontology tokens. The number of errors was also increased proportionally with the length of the tokens:

- for lengths less than 10, we introduce one error,
- for lengths of 10–17, we introduce three errors,
- for all other lengths, we introduce four errors.

This resulted in a total of 38 108 unique typographical errors. This list was used to systematically replace “canonical” tokens in randomly chosen ontological concepts containing them, which resulted in 38 108 unique ontology labels. For example, the “canonical” token acylglycine in HP:0012073 (“Abnormal urinary acylglycine profile”) was used to generate 24 new labels using 24 typographical errors, such as: “Abnormal urinary ackylglycine profile,” “Abnormal urinary acfydlrglycine profile,” or “Abnormal urinary aclgycine profile.” These labels were then used as input text in our experiments discussed in Section 3.2.

## 3 Results

We performed a comprehensive evaluation of our approach, in conjunction with five state-of-the-art HPO CR tools discussed in Section 2.8.1.

### 3.1 Assessment of CR performance

CR, as a task, is conceptually composed of two parts: boundary detection and entity linkage. Boundary detection refers to finding the spans of text that represent candidate-named entities, while entity linkage—given a target ontology—focuses on finding the best concept that matches the candidate-named entity. The alignment strategy proposed in this article is fit for this second component, and as such, in a practical scenario would require candidates to be provided as input. Consequently, the validation and results discussed here start from a set of potential named entity candidates and record the ability of finding the best matching HPO concept.

From a process perspective, we used the text spans corresponding to the manual annotations recorded in the corpora introduced in Section 2.8.2 to create fake sentences—e.g. the text span “*dysplastic hip joints*” was converted to “*Dysplastic hip joints.*” These were then provided to the tools listed in Section 2.8.1—in addition to ours—as input candidates for matching. All HPO IDs resulted from the annotations produced by the tools were aligned to HPO version 02–2022 using the same procedure as for the GSC+ corpus. Moreover, in order to ensure a fair comparison of the results, the evaluation metrics considered only the children of HP:0000118 (*Phenotypic abnormality*), since PhenoTagger was trained to perform CR on this subset of HPO.

To ensure, we record the best performance of the tools, in some cases, we ran several experiments using different forms of the corpora. In particular, we wanted to emphasize the difference made by using the full-text entries versus named entities for approaches relying on BERT models, where the lack of context could negatively influence the annotation outcome. Consequently, as presented in the next section, PhenoTagger and ClinPheno were also run in baseline on the complete abstracts. To be able to produce results with Doc2HPO, we created well-formed sentences. Finally, due to the sensitive nature of the content, the EHR corpus was analyzed only with tools available in offline mode—i.e. PhenoTagger and ClinPheno.

We used the standard metrics for evaluating named entity or CR performance: Precision, Recall, and F1. Precision is defined as the ratio between the correctly identified positives (true positives) and all identified positives. Recall is the ratio between the predicted true positives and the actual annotation outcomes produced by the tool. F1 = 2 × Precision × Recall/(Precision + Recall).

### 3.2 Experimental results

Table 5 lists the results achieved by all tools, as well as exact matching on the GSC+ (baseline) and EHR corpora (in exact matching, a hit is recorded when the compared tokens are equal). Their results serve two purposes. Firstly, we wanted to establish a sense of performance when the tools are applied in the absence of typographical errors. Overall, the difference in F1 score between the top-scoring approaches was roughly 7% (PhenoTagger—full text versus ClinPheno), and the maximal difference was 24% (PhenoTagger—full text versus ClinPheno—full text). It is important to note the significantly higher recalls achieved by PhenoTagger and ClinPheno on the manually annotated concepts in the two corpora. Secondly, in the case of PhenoTagger and ClinPheno, we intended to capture the difference made by using full text versus named entities as input. While for PhenoTagger we observe a difference of 5% in F1, ClinPheno performed significantly better on named entities than on full text. These results validate the outcomes discussed below on typographical errors.

**Table 5.** Experimental results achieved on baseline corpora.^a^

	GSC+			EHR		
Method	P	R	F1	P	R	F1
Exact matching	94.2	42.6	58.6	99.8	58.0	**73.4**
PhenoTagger	67.3	66.3	66.8	68.3	67.9	68.1
PhenoTagger (full text)	77.0	*67.9*	**72.2**	59.9	*70.0*	64.6
ClinPheno	63.7	65.3	64.5	60.3	62.7	61.4
ClinPheno (full text)	73.2	36.0	48.3	45.8	52.5	48.9
Doc2HPO	80.5	49.8	61.5			
Monarch Annotator	82.3	50.2	62.3			
NCBO Annotator	66.0	49.1	56.3			

a Bolded values represent the best F1 in class, while italics denote the best recall.

As presented in Section 2.8.2, the results listed in Table 6 were achieved by introducing typographical errors to replace “canonical” tokens present in ontological concepts. As baseline, unsurprisingly, the Exact matching approach records a close to null recall. This is, however, considerably improved by using our alignment strategy with optimal score cut-offs—a jump from 0.3% to 24.7% on GSC+ and from 0.4% to 40.7% on EHR. The results of the other approaches show clearly that, with the exception of PhenoTagger, they cannot handle typographical errors appropriately, and as such, they could lead to false negatives when applied in a clinical context.

**Table 6.** Experimental results achieved on typo corpora listed in Table 4.

	GSC+			EHR		
Method	Precision	Recall	F1	Precision	Recall	F1
Exact matching	93.3	0.3	0.7	80.3	0.4	0.8
PhenoTagger	34.6	17.3	23.1	58.8	19.4	29.2
ClinPheno	28.8	12.3	17.2	43.2	17.2	24.6
Doc2HPO	34.1	7.7	12.6			
Monarch Annotator	12.1	3.2	5.1			
NCBO Annotator	12.5	3.32	5.2			
TBLAT	96.0	24.7	**39.3**	98.5	40.7	**57.6**

Bolded values represent the best F1 score in class.

Finally, Table 7 lists the results achieved on the second corpus (GenTypo) by the top scoring approaches on GSC+ (typo). While Doc2HPO and ClinPheno recorded a significant drop in performance, PhenoTagger produced consistent results, and furthermore, recorded a significant increase in recall to 26.4%. On a different note, our alignment strategy achieved the best performance with a recall of 73.2%. It is worth reiterating that the GenTypo corpus covered tokens not seen or used in establishing the score distributions and cut-off, and as such, they can be considered a valid test set.

**Table 7.** Experimental results achieved on GenTypo.

Method	Precision	Recall	F1
PhenoTagger	52.5	26.4	35.1
Doc2HPO	4.4	0.6	1.0
ClinPheno	5.6	1.2	2.0
TBLAT	96.3	73.2	**83.2**

Bolded value represents the best F1 score in class.

### 3.3 Error analysis

A closer look at the errors produced by our alignment strategy on the typo corpora revealed that 86.2% were the same HPO entries not found by the exact matching approach, which is unsurprising since even a correct alignment would not have led to a correct entity linkage. 1.4% of the errors led to an incorrect alignment, which then led to an incorrect entity linkage. The rest of the 12.4% errors were associated with a lack of or an incorrect alignment, which led to no entity linkage.

Over 85% of the incorrectly aligned tokens were of length six or less, with single replacement and gap shift errors, combined with the lack of the addition of a single character, dominating the pool. Prominent examples include: *tugor* →*rigors* instead of *tumor*; *spnal* →*spinal* instead of *renal*; *rernal* →*renal* instead of *sternal*; *reinal* →*renal* instead of *spinal*. Similarly, examples of tokens failed to be aligned included: *nsaiss* and *nagls* →*nails* or *thrumbs* and *thumns* →*thumbs*. The alignment of longer tokens was affected by the presence of multiple simultaneous types of errors. Examples include: *annomaliss* and *anolamies* →*anomalies*. The same patterns were present in the GenTypo corpus evaluation, with the recall achieving a higher value because of the significantly larger diversity of tokens and ontology concepts when compared to the typo corpora. The errors we introduced were also less complex. They were, however, problematic enough to showcase the challenges standard phenotype concept recognizers would to address in a real-world clinical environment.

In conclusion, the challenges we observed were related to the alignment of short tokens, where every change can have a significant impact, and of long tokens in the presence of complex typographical errors.

## 4 Discussion

The results discussed in the previous section show that our alignment strategy is generalizable to unseen tokens, as well as tokens of lengths over 10. While our focus has been on phenotypes and the HPO, in a clinical care context, the algorithm can be similarly employed using other health-related ontologies or subontologies, such as SNOMED CT or ICD. Moreover, its applicability is not limited to symptoms/phenotypes, but rather can be extended to diseases (e.g. Disease Ontology or Orphanet Rare Disease Ontology), drugs (e.g. DrugBank), or interventions (e.g. Medical Action Ontology)—all of which cover medical concepts that are susceptible to typographical errors.

As a limitation, it is, however, dependent on the type and number of typographical errors. While we have attempted to classify and describe most types of typographical errors, more than 20% of our corpus remained uncategorized. Most of these entries represent a combination of errors that increase the complexity of the alignment. The length of the target tokens also plays an important role in a successful alignment. Unsurprisingly, shorter tokens were harder to reconcile, due to the small number of 3-mers. Moreover, every character change in the target token leads to a change in at least one 3-mer (if the change occurs at the very beginning of very end) and usually three 3-mers (if the change occurs somewhere else in the token).

On a different note, the main driver behind the proposed method was the applicability in a clinical setting. Our view on applicability covers two aspects. Firstly, the HPO CR had to perform well in the presence of typographical errors, and as shown in Section 3.2 our alignment strategy delivered satisfactory results. Secondly, most clinical environments are still resource-constrained—e.g. they use desktop PCs or NUCs, which are not equipped for data-intensive tasks. Tools such as PhenoTagger (the state-of-the-art performer) rely on deep learning models and require appropriate resources to run (more concretely, at a minimum 3GB dedicated RAM to load the model in memory). Our alignment strategy, however, can be coupled with an arbitrary (less resource-intensive) concept recognizer to improve performance on typographical errors without introducing additional computational overhead. The algorithm itself requires a fraction of a single CPU to run and has a memory footprint of 1.2MB for the scoring matrix + ∼1 MB to store the list of all unique tokens in HPO. It also does not require any additional setup or access to external resources. In terms of complexity, for a given token t, the time complexity associated with computing the alignment score is *O(nk)*, where *k* is t’s number of 3-mers (i.e. *len(t) − 2*) and *n* is the number of candidate ontology concept unigrams. *n* can be reduced to a worst-case scenario of *log(n)* subject to the candidate selection strategy, which in practice would be driven by t’s 3-mers.

## 5 Conclusion

The aim of this article was to perform a systematic analysis of the impact of typographical errors on phenotype CR. We have introduced a gold standard of typographical errors collected from a real-world clinical setting and used the corpus to showcase the complexity of the domain. All state-of-the-art tools encountered significant difficulties in dealing with the types of errors populating the gold standard. In addition, we proposed a lightweight bioinformatics-inspired alignment strategy that can be used to augment any of the existing methods and boost their CR performance. The experimental results showed a significant improvement in recall on both an existing corpus, as well as one generated specifically for the purposes of our evaluation.

## Supplementary Material

btad716_Supplementary_Data
