# Supplementary material for: Term-BLAST-like alignment tool for concept recognition in noisy clinical texts
Source: Bioinformatics. 2023 Nov 24;39(12):btad716. doi: 10.1093/bioinformatics/btad716 (PMC10710372; doi:10.1093/bioinformatics/btad716)

Term-BLAST-Like Alignment Tool for Concept Recognition in Noisy Clinical Texts
- Supplementary material -

1. List of stop words removed by the TBLAT algorithm

- a, the, and, of, in, to, on, an, with

2. Coverage of the typographical errors associated with the ontological tokens in the gold standard given the underlying score distribution and group according to the length of the token.

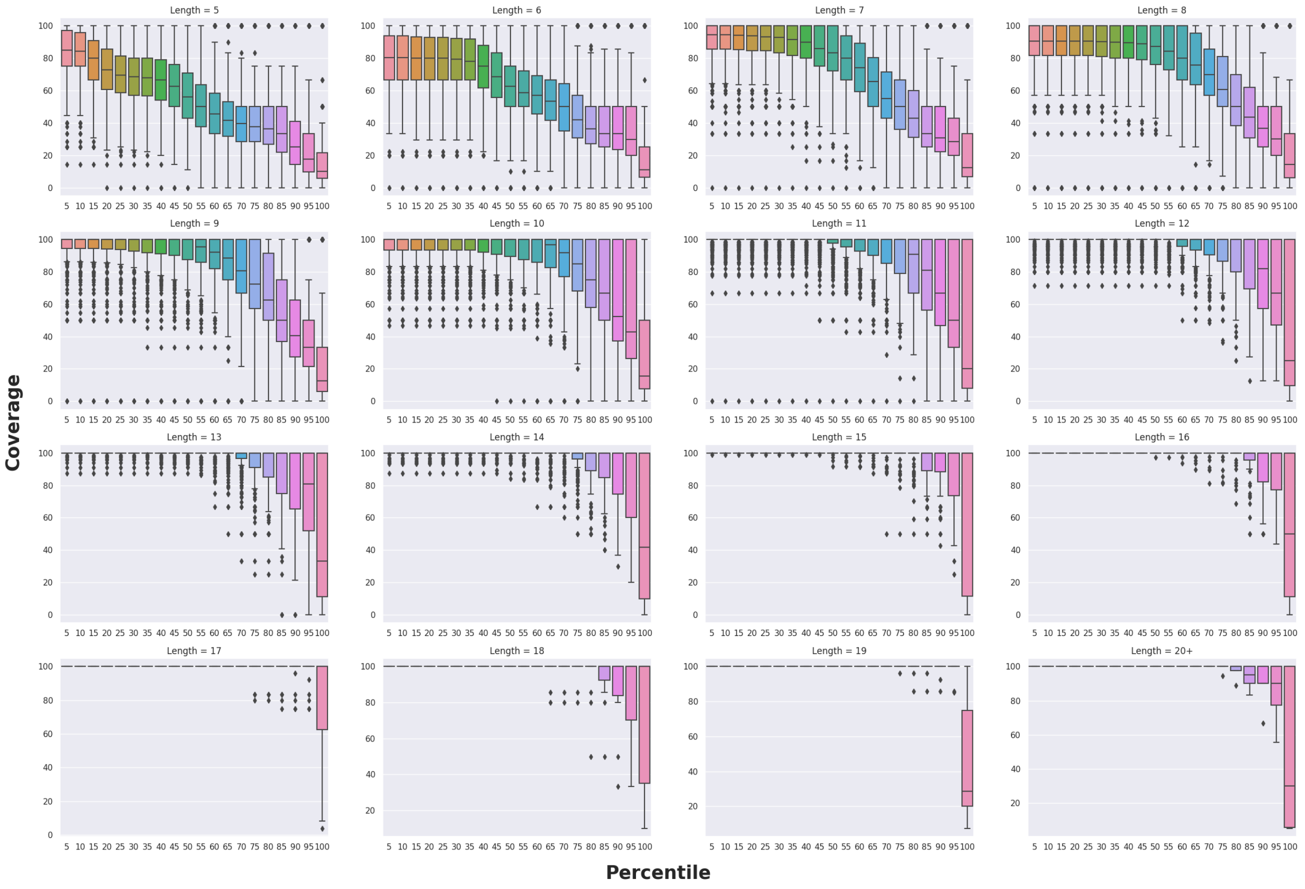

Supplement: btad716_Supplementary_Data [file btad716_supplementary_data.pdf]
